# Recurrent Nephrolithiasis Due to Parathyroid Adenoma

**DOI:** 10.7759/cureus.18468

**Published:** 2021-10-04

**Authors:** Gyanendra Bagale, Sandip R Pradhan, Archana Basnet

**Affiliations:** 1 Otorhinolaryngology, Patan Academy of Health and Sciences, Kathmandu, NPL; 2 Internal Medicine, Lumbini Medical College, Palpa, NPL; 3 Medicine, De la Salle Health Sciences Institute, Cavite, PHL

**Keywords:** renal stone, nephrolithiasis, hyperparathyroidism, parathyroid adenoma, hypercalcemia

## Abstract

Nephrolithiasis is a common urologic disorder affecting various age groups worldwide, in association with significant morbidity. The cause associated with renal stone varies, of which increased calcium level due to primary hyperparathyroidism (parathyroid adenoma) is one of the unique and rare causes associated. The case report we are presenting is a 35-year-old female with a history of recurrent renal stones in developing country settings. Upon detailed workup, parathyroid adenoma hence was identified to be the primary culprit. She underwent parathyroidectomy and has recovered well without any complications.

## Introduction

Nephrolithiasis (also referred to as urolithiasis) is a common worldwide urological disorder causing significant morbidity and increased health expenditure. In industrialized countries, up to 12% of men and 7% of women will form a kidney stone in their lifetime, and the prevalence appears to be increasing [1]. The presentation may be symptomatic or asymptomatic. It may be due to calcium stones (calcium oxalate and calcium phosphate) which comprises approximately 80% of all the stones in addition to uric acid about 9%, and struvite (magnesium ammonium phosphate hexahydrate) about 10%, leaving only 1% for all the rest (cystine, drug stones, ammonium acid urate) [2].

The common causes associated with renal stones are high urine calcium, high urine oxalate, high urine uric acid, and low urine citrate, glucocorticoids, loop diuretics, antivirals like acyclovir, ritonavir, primary hyperparathyroidism, obesity, distal renal tubular acidosis, cystinuria, urinary tract infection, familial history, genetic and environmental factors [3]. Among listed factors, nephrolithiasis is a common and classic renal manifestation of primary hyperparathyroidism. Approximately about 20% of individuals with primary hyperparathyroidism have renal stone occurrence [4]. On the contrary, approximately 5% of individuals with renal stones have hyperparathyroidism [5].

Nephrolithiasis also carries a higher recurrence rate after the initial episode. The recurrence rates per 100 person-years were 3.4 after the first episode, 7.1 after the second episode, 12.1 after the third episode, and 17.6 after the fourth or higher episode [6]. In addition, retrospective studies have shown the “natural cumulative recurrence rate of stones” to be 14% at one year, 35% at five years, and 52% at ten years [7].

## Case presentation

A 35-year-old female presented to the clinic with a complaint of dull aching pain in the left flank region with occasional sharp episodes and no radiation. She had no history of fever, burning micturition, hematuria, and increased urinary frequency and urgency. She complained of occasional fatigue but denied nausea, weight loss, blurred vision, headache, neck swelling, excessive thirst, constipation, muscle and joints pain, mood changes, amenorrhea, and galactorrhoea.

She had a similar history of nephrolithiasis episodes in the past and had undergone left and right percutaneous nephrolithotomy (PCNL) surgery 15 and 10 years back, respectively. In addition, she had no family history of similar illnesses in other family members. She denied tobacco consumption but consumed alcohol occasionally.

Due to similar previous episodes, further studies were done revealing elevated serum calcium and parathyroid hormone (PTH) with 11.5 mg/dl (normal range: 8.8-10.5mg/dl) and 152.6 (normal range: 7.5-53.5pg/ml), respectively. The level of vitamin D was 22.7 ng/ml and was slightly in the level of the lower range. In addition, she had normal 24 hours urinary calcium level, prolactin, serum albumin, magnesium, phosphorus, lactate dehydrogenase, thyroid function test (TFT), and urine examination.

**Figure 1:**
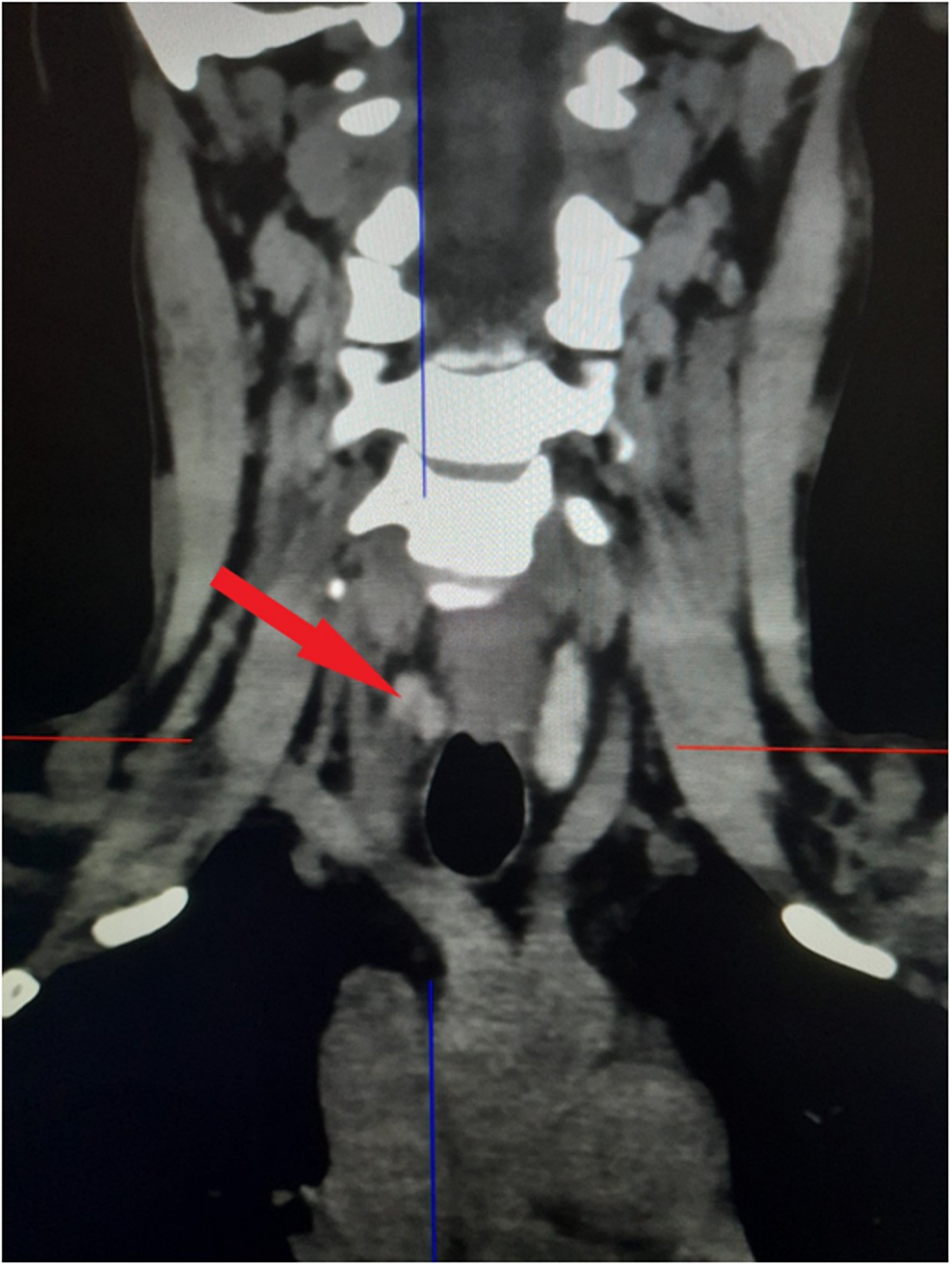
Four-dimensional computed tomography showing parathyroid adenoma (red arrow).

**Figure 2:**
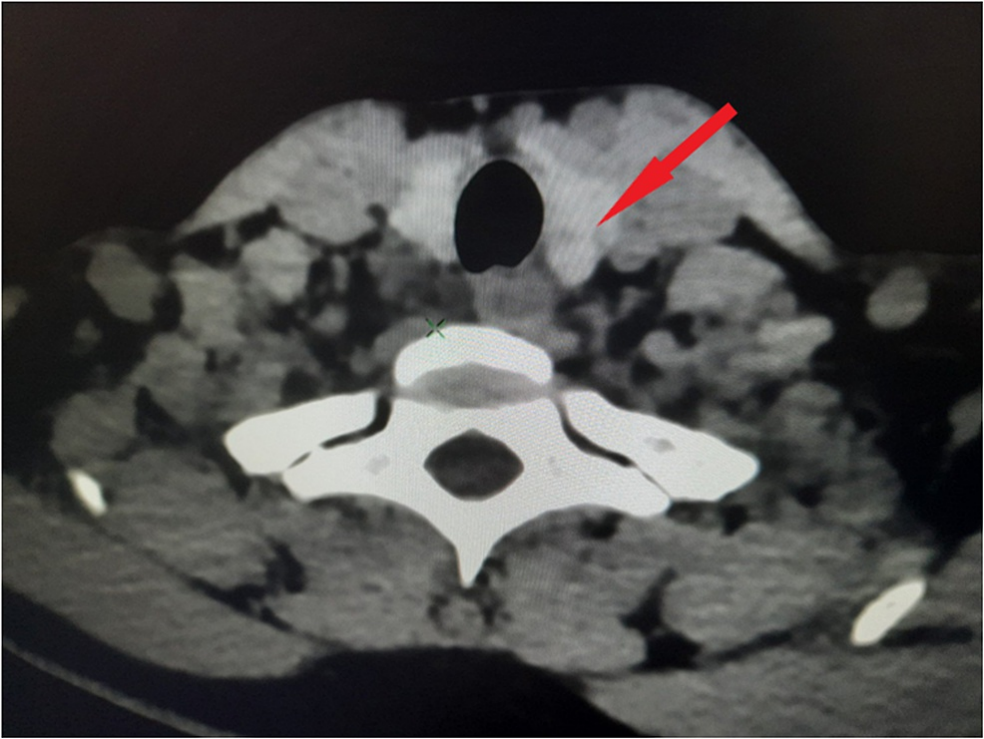
Four-dimensional computed tomography scan showing parathyroid adenoma (red arrow).

As part of the protocol, an ultrasound of the neck was solicited but was unexceptional. Hence, a 4D CT scan of parathyroid was commenced, which showed soft tissue density lesion at the posterior aspect of the lower pole of the right lobe of thyroid with hyperenhancement in arterial phase and washout in venous phase compared to the thyroid, and an inferior thyroid artery as a polar vessel - features suggestive of parathyroid adenoma (Figures 1-2).

**Figure 3:**
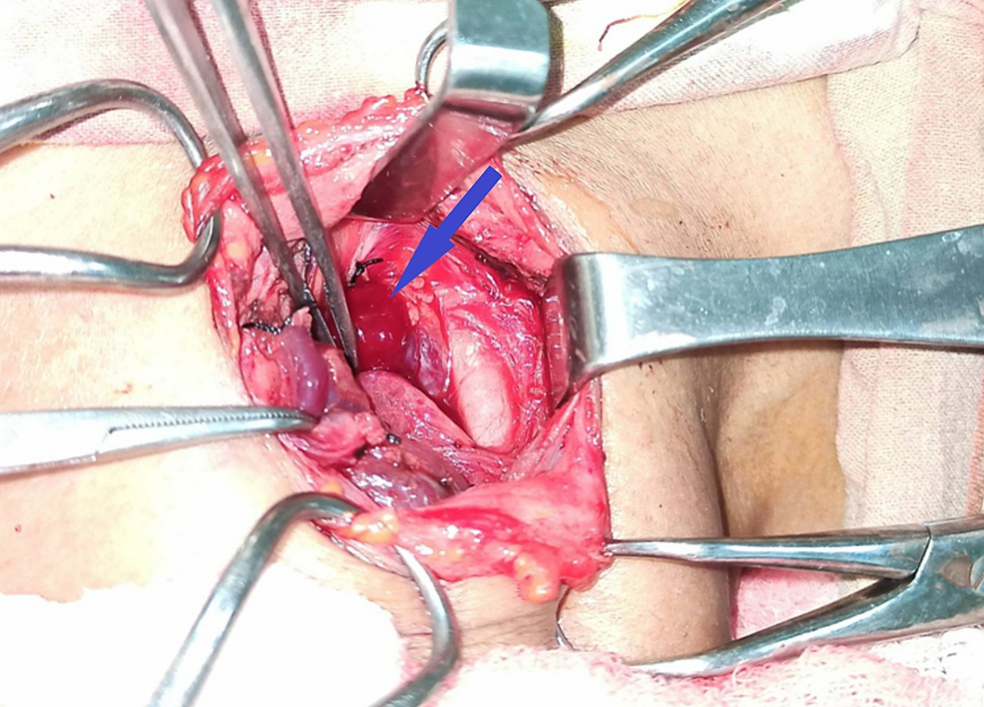
During surgery, parathyroid adenoma identified as reddish mass (blue arrow)

**Figure 4:**
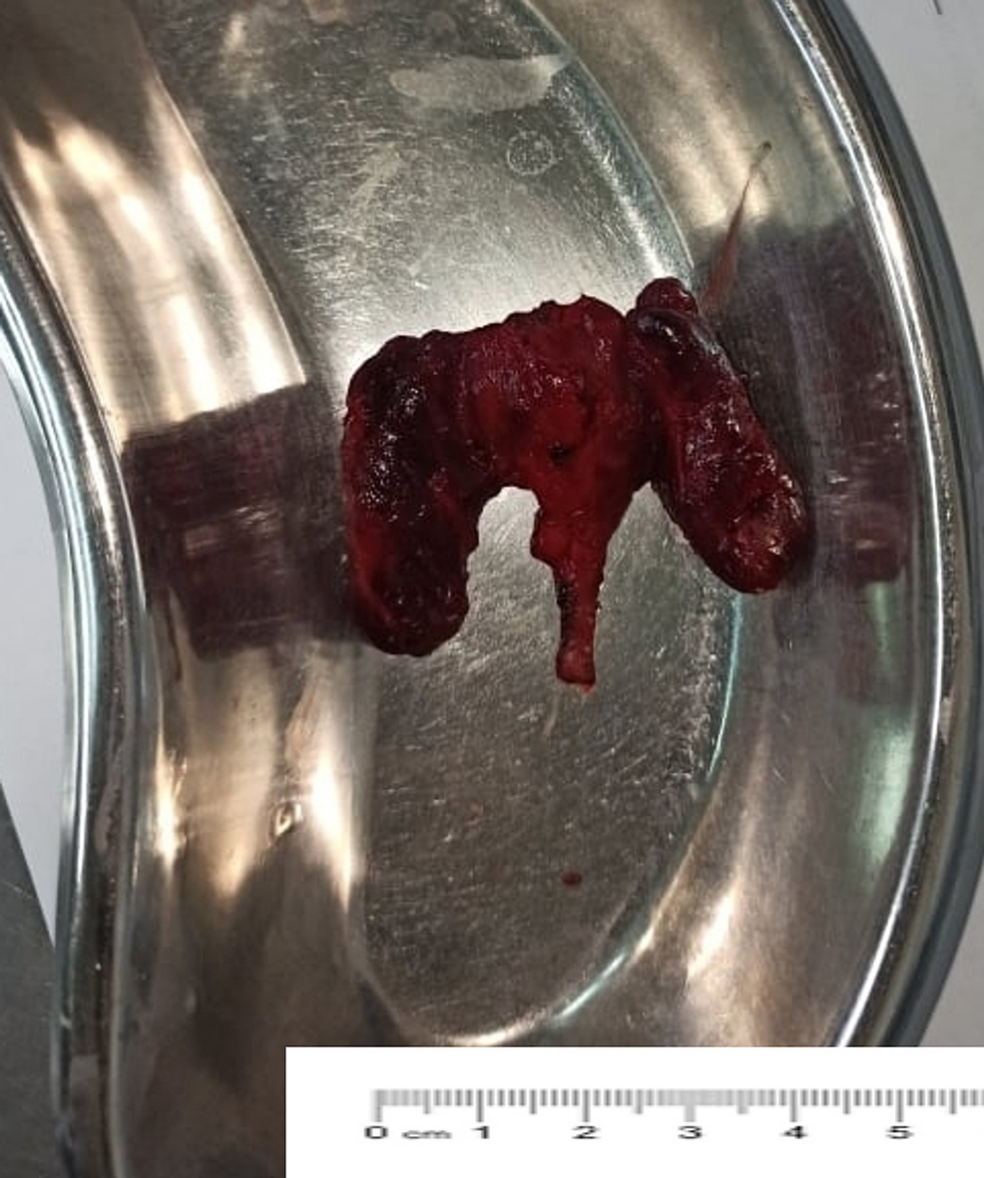
Parathyroid adenoma measuring 4 cm × 2 cm after surgical removal

Later, Sestamibi parathyroid scintigraphy confirmed the right parathyroid adenoma. The bone mass density (BMD) was also performed which revealed normal spine and femur density. Hence, she was diagnosed with recurrent nephrolithiasis secondary to hypercalcemia due to primary hyperparathyroidism. As a result, she underwent Right inferior focused parathyroidectomy (Figures 3-4).

The level of PTH one hour before the surgery was 187.2 pg/ml, and the level of PTH 20 minutes after the surgery was 21.6 pg/ml, which decreased significantly. However, PTH and corrected calcium levels were normal (21.5 pg/ml and 10.6 mg/dl, respectively) on the subsequent second postoperative day. She had recovered well after the surgery, and currently, she is in good health with no complications. She is on regular follow-up.

## Discussion

Primary hyperparathyroidism is an endocrine pathology that affects calcium metabolism. Primary hyperparathyroidism is due to the presence of a parathyroid adenoma in 80-85% and multiple gland hyperplasia in 10-15% cases [8]. Primary hyperparathyroidism is the third most common endocrine disorder. The prevalence is between 27 and 30 per 100 000 individuals [9]. In addition, there is a four to eightfold increased prevalence of nephrolithiasis in patients with primary hyperparathyroidism than subjects not affected by the disorder [10,11].

The exact pathogenesis of stone formation remains unclear. Hypercalciuria is a common finding in primary hyperparathyroidism and has been implicated in the formation of nephrolithiasis. Parathyroid hormone increases the synthesis of calcitriol, which increases the intestinal absorption of calcium. Parathyroid hormone also raises renal tubular calcium reabsorption as well as the rate of bone turnover. Increased serum parathyroid hormone, therefore, causes hypercalcemia and hypercalciuria that increases the supersaturation of calcium oxalate in the urine resulting in the formation of stone [12]. Patients with primary hyperparathyroidism have a greater risk of nephrolithiasis than a population-based control group matched for sex and age, even ten years before the diagnosis was registered. So it suggests that the condition has started several years before diagnosis and emphasizes the importance of early diagnosis and treatment [13].

Parathyroidectomy is associated with an 8.3% risk reduction in nephrolithiasis events, and more than ten years after surgery, the risk returns to that of controls [13]. The study shows about 1.5% of recurrence in symptomatic recurrent nephrolithiasis following parathyroidectomy in patients with a prior history of nephrolithiasis formation compared to idiopathic stone formation, of whom 25% experienced recurrence [14]. There is no sign of recurrence in our patient after one year of surgery.

## Conclusions

Nephrolithiasis is a ubiquitous urological disorder of multifactorial origin, causing increased morbidity and health expenditure. Primary hyperparathyroidism should also be suspected as one of the primary causes of recurrent nephrolithiasis. The mainstay of treatment is parathyroidectomy, thus, decreasing the recurrence rate of nephrolithiasis.
